# Tired People

**Published:** 1892-08

**Authors:** 


### TIRED PEOPLE.

Of the many unfortunate people in the world, there are few so deserving of pity as those whose daily toil is such a hardship to them that neither strength nor inclination is left for anything else. The drain upon their energies is so heavy that nature is unable to meet it; and now and then there is a break down, which means loss of work and of money, and a doctor's bill into the bargain. To such sufferers we would suggest the advisability of looking at matters fairly and thoroughly, and asking oneself whether it is not possible to lighten in some measure the burden of work and responsibility that is now crushing the zest and joy out of life. Some of us have got into the uncomfortable habit of doing things we have no business to do. In our anxiety to have the work well done we go fidgetting round, giving finishing touches that are not needed, and allowing it to absorb more time and strength than we can spare. Then, again, that happy knack that some persons have of working quietly, easily, and with as much contentment as can be gained out of the day's experience, is worth while cultivating, for the sake of the physical exhaustion it will save. A bustling, worrying, excitable workman goes through far more exertion, yet does his duty no better, than his more orderly neighbor. The old saying that "it's no use killing yourself to keep yourself," has a grain of wisdom in it for tired people. The most effectual remedy for the weariness that proves so embarrassing in the majority of instances is to lessen the present strain somehow, and be careful not to add new unnecessary burdens. When nature calls for a rest, her demand must be obeyed, and it must be a genuine respite from toil. There is not much permanent advantage to be derived from trying to push a number of little jobs in edgeways, "while we are resting," and a persistent adherence to such a practice will result, sooner or later, in a weariness of body and a depression of spirit that will tax to their utmost the best efforts of doctors and nurses.—*G. Y., in Health and Home, Eng.*

